# Characterization of Diarrheagenic Strains of *Escherichia coli* Isolated From Cattle Raised in Three Regions of Mexico

**DOI:** 10.3389/fmicb.2018.02373

**Published:** 2018-10-11

**Authors:** Armando Navarro, Patricia Isidra Cauich-Sánchez, Alejandro Trejo, Alvaro Gutiérrez, Sylvia Paz Díaz, Martha Díaz C., Alejandro Cravioto, Carlos Eslava

**Affiliations:** ^1^Department of Public Health, Faculty of Medicine, Universidad Nacional Autónoma de México, Mexico City, Mexico; ^2^Departamento de Microbiología, Escuela Nacional de Ciencias Biológicas, Instituto Politécnico Nacional, Mexico City, Mexico; ^3^Universidad de Guadalajara, Guadalajara, Mexico; ^4^Laboratorios Veterinarios Halvet SA de CV, Universidad de Guadalajara, Guadalajara, Mexico; ^5^Unidad de Investigación en Salud Pública Kaethe Willms, Facultad de Ciencias Químico Biológicas, Universidad Autónoma de Sinaloa, Culiacán, Mexico; ^6^Departamento de Ciencias Químico Biológicas, Universidad de Sonora, Hermosillo, Mexico; ^7^Faculty of Medicine, Universidad Nacional Autónoma de México, Mexico City, Mexico; ^8^Peripheral Unit of Basic and Clinical Research in Infectious Diseases, Bacterial Pathogenicity Laboratory, Hemato Oncology and Research Unit, Department of Public Health/Research Division Medicine Faculty, Children’s Hospital of Mexico Federico Gómez, National Autonomous University of Mexico, Mexico City, Mexico

**Keywords:** *Escherichia coli*, serogroups, serotypes, DEC, non-O157 STEC, phylogenetic groups

## Abstract

Intestinal infections represent an important public health concern worldwide. *Escherichia coli* is one of the main bacterial agents involved in the pathogenesis of different diseases. In 2011, an outbreak of hemorrhagic colitis (HC) and hemolytic uremic syndrome (HUS) in Germany was related to a non-O157 STEC strain of O104:H4 serotype. The difficulty in identifying the origin of the bacteria related to the outbreak showed the importance of having epidemiological information from different parts of the world. The aim of this study was to perform a retrospective analysis to determine if *E. coli* strains isolated from cattle from different locations in Mexico have similar characteristics to those isolated in other countries. Samples obtained in different years from 252 cows belonging to 5 herds were analyzed. A total of 1,260 colonies were selected from the 252 samples, 841 (67%) of which corresponded to *E. coli* and 419 (33%) to other enterobacteria. In total, 78% (656) of the *E. coli* strains could be serotyped, of which 393 (59.9%) belonged to 5 diarrheagenic (DEC) pathotypes. Serotyping showed STEC (40.7%) and ETEC (26.7%) strains were more common. PCR assays were used to determine the presence of STEC (*eae*, *stx1*, *stx2*, and *ehxA*) and EAEC (*aatA*, *aggR*, and *aapA*) genes, and phylogenetic groups. The results showed that 70 strains belonging to 23 serogroups were *stx1* and *stx2* positive, while 13 strains from the O9 serogroup were *ehxA*, *aggR*, and *eae* positive. Phylogenetic analysis showed 58 (82.9%) strains belonged to A and B1 commensal phylogroups and 12 (17.1%) to B2, D and E virulent phylogroups. An assay to evaluate cross-antigenic reactivity in the serum of cattle between K9 capsular antigen and O104 LPS by ELISA showed similar responses against both antigens (*p* > 0.05). The antimicrobial sensitivity assay of the strains showed resistance to AM, CEP, CXM, TE, SXT, cephalosporins and fluoroquinolones. The results show that cattle are carriers and potential transmitters of STEC and ETEC strains containing virulence genes. Epidemiological retrospective studies in different countries are of great help for identifying virulent bacterial strains with the potential to cause outbreaks that may have epidemiological impact in susceptible countries.

## Introduction

*Escherichia coli* (*E. coli*) strains continue to be a frequent cause of human infection. These infections can be restricted to the gastrointestinal track or the cause of urinary, systemic or meningeal infections (Croxen et al., 2013). In the case of gastrointestinal infections, *E. coli* strains associated with disease have been classified by their virulent characteristics into at least six different pathogenic clusters that constitute the so-called Diarrheagenic *E. coli* group (DEC) (Kaper et al., 2004). Human feces constitute the most frequent source of DEC strains, either through direct hand-to-mouth transmission or by the contamination of water and food (Qadri et al., 2005; Chekabab et al., 2013; Luna-Gierke et al., 2014). Animal feces are a secondary source of human infection for some DEC strains, mainly those capable of producing Shiga-like toxin. These group of DEC strains have been called Shiga toxin-producing *E. coli* (STEC) and represent the most frequent cause of severe hemorrhagic colitis (HC) and hemolytic-uremic syndrome (HUS), since their discovery in the early 80 s (Karmali et al., 1983; Riley et al., 1983). While most of the large outbreaks of HC and HUS have been associated with specific STEC belonging to the O157:H7 serotype, STEC strains belonging to other O:H serotypes have also been linked to HC and HUS (Mead et al., 1999; Brooks et al., 2005; Gould et al., 2013; Luna-Gierke et al., 2014). This latter group includes the recently isolated O104:H4 strains, which were associated with an important outbreak of HC and HUS in Germany (Frank et al., 2011). These outbreak strains represent a hybrid with virulence traits from both STEC and Enteroaggregative *E. coli* (EAggEC) strains, which is something that had not been reported previously (Scheutz et al., 2011). Given our interest in the frequency of DEC strains as a cause of diarrheal disease in Mexican children over the past 30 years and the capacity of our laboratory to conduct full serotyping of O and H antigens, and the determination of the virulence characteristics of putative DEC strains isolated from both humans and animals, we describe here the frequency and characteristics of *E. coli* strains isolated from cattle raised in three different regions of Mexico and discuss the capacity of these strains to cause human disease outbreaks.

## Materials and Methods

### Biological Material

The protocol was approved by the Research Committee (IN225705) of the Faculty of Medicine at the National Autonomous University of Mexico. Sampling of dairy cows was conducted in accordance with specific techniques for the production, care and use of laboratory animals, as described in the Mexican Official Norm 062-Zoo-1999 (NOM-062-Zoo-1999; NORMA Oficial Mexicana, 1999).

Fecal, rectal and blood samples were collected from 252 cows belonging to five herds of lactating cows raised in Jalisco (Jal), Sinaloa (Sin), and Sonora (Son), Mexico. Sampling of the dairy cows was carried out in three different years (2004, 2005, and 2007). Fecal samples (10 g) were collected from the rectum of the cattle. Following collection, cotton swabs were dipped into Cary-Blair transport medium and the samples were kept at 4°C until they reached the laboratory for processing within 3 h of arrival.

### Bovine Serum Samples

Serum samples were obtained from total blood taken from the coccygeal vein of the cows, after cleaning and disinfecting the area with soap, water and alcohol (70%). Each blood sample was obtained using a 5 mL syringe with a 22G needle. Approximately 3 mL of blood was taken and placed in a Vacutainer tube without anticoagulant (BD). As with the fecal samples, the blood samples were kept under refrigeration at 4°C until they reached the laboratory for processing. Serum separation was performed in the laboratory and kept until use at -20°C in 100 μL aliquots.

### Isolation and Identification of Strains

For the isolation of *E. coli* and other *Enterobacteriaceae*, the fecal samples were streaked onto MacConkey agar with lactose and sorbitol and the plates were incubated overnight at 37°C. For the isolation of antigenic representative varieties of *E. coli* and other enterobacteria, five or more isolated colonies were selected from the agar plates. The cultures were identified separately using the GNI card (Gram-negative identification) of the automated VITEK2 system (compact model, bioMérieux).

### Serotyping

Strains identified as *E. coli* were serotyped by agglutination assays using 96-well micro titer plates and rabbit antisera against O1 to O187 somatic (O) antigens and 53 flagellar (H) antigens prepared in rabbits (SERUNAM, registered trademark in Mexico, with number 323158/2015) using the method described by Orskov and Orskov (1984) with minor modifications.

### Virulence Genes

Virulence genes for Shiga-toxin producing *E. coli* (STEC) and enteroaggregative *E. coli* (EAEC) were detected by PCR using the primers described in **Table 1** under conditions previously described (Schmidt et al., 1994, 1995; Czeczulin et al., 1999; Cerna et al., 2003; Nishi et al., 2003; Scheutz et al., 2012).

**Table 1:** Primers used to identify virulence genes and phylogenetic groups.

Genes	Code	Sequence (5′ a 3′)	Product size (pb)	Reference
*eae* (Universal)	Sk1-F	CCCGAATTCGGCACAAGCATAAGC	864	Schmidt et al., 1994
	Sk2-R	CCCGGATCCGTCTCGCCAGTATTC		
*stx1*	vtx1-F	GTACGGGGATGCAGATAAATCGC	209	Scheutz et al., 2012
	vtx1-R	AGCAGTCATTACATAAGAACGYCCACT		
*stx2*	vtx2-F	GGCACTGTCTGAAACTGCTCCTGT	627	
	vtx2-F	ATTAAACTGCACTTCAGCAAATCC		
*ehxA*	ehxA-F	GGTGCAGCAGAAAAAGTTGTAG	1551	Schmidt et al., 1995
	ehxA-F	TCTCGCCTGATAGTGTTTGGTA		
arpA	arpA-F	AACGCTATTCGCCAGCTTGC	400	Clermont et al., 2013
	arpA-R	TCTCCCCATACCGTACGCTA		
chuA	chuA-F	ATGGTACCGGACGAACCAAC	288	
	chuA-R	TGCCGCCAGTACCAAAGACA		
YjaA	yjaA-F	CAAACGTGAAGTGTCAGGAG	211	
	yjaA-R	AATGCGTTCCTCAACCTGTG		
TspE4.C2	TspE4-F	CACTATTCGTAAGGTCATCC	152	
	TspE4-R	AGTTTATCGCTGCGGGTCGC		
aatA	aatA-F	ATGTTACCAGATATAAATATAG	1064	Nishi et al., 2003
	aat-R	CATTTCCCCTGTATTGGAAATG		
*aggR*	aggR-F	CTAATTGTACAATCGATGTA	308	Czeczulin et al., 1999
	aggR-R	ATGAAGTAATTCTTGAAT		
*aapA*	aapA-F	CTTGGGTATCAGCCTGAATG	310	Cerna et al., 2003
	aapA-R	AACCCATTCGGTTAGAGCAC		

### Phylogenetic Grouping

Phylogenetic groups were determined by PCR multiplex using the primers described in **Table 1** and under conditions detailed by Clermont et al. (2013). The designation of the phylogenetic groups (A, B1, B2, C, D, E, F, and *Escherichia* cryptic clade I) was carried out by the presence or absence of *chuA*, *yjaA*, TspE4.C2 and the *arpA* gene using a flow scheme (Clermont et al., 2013).

### Antimicrobial Resistance

Antimicrobial susceptibility testing was performed according to the manufacturer’s instructions (bioMérieux). In brief, antimicrobial resistance testing was performed by the VITEK2 automated system (compact model, bioMérieux). Each strain was inoculated into nutrient agar (pH 7.4) and incubated overnight at 37°C. The growth of each culture was diluted in 2.0 mL of 4.5% sterile saline solution and then adjusted to tube number 1 of McFarland nephelometer (3 × 10^8^ bacteria/mL). The bacterial suspension was then inoculated by the automated system onto an AST-GN23 card to test its reaction against antimicrobial agents for gram negative organisms.

The concentration of antimicrobials in the AST-GN23 card was as follows: amoxicillin/clavulanic acid (AMC) 4/2, 16/8, 32/16 μg/mL; ampicillin (AM) 4, 8, 32 μg/mL; cephalothin (CEP) 2, 8, 32 μg/mL; cefazolin (CZ) 4, 16, 64 μg/mL; cefepime (FEP) 2,8, 16, 32 μg/mL; cefoxitin (FOX) 8, 16, 32 μg/mL; cefpodoxime (CPD) 0.5, 1, 4 μg/mL; ceftazidime (CAZ) 1, 2, 8, 32 μg/mL; ceftriaxone (CRO) 1, 2, 8, 32 μg/mL; cefuroxime (CXM) 2, 8, 32 μg/mL; ciprofloxacine (CIP) 0.5, 2, 4 μg/mL; gentamicin (GM) 4, 16, 32 μg/mL; levofloxacin (LEV) 0.25, 0.5, 2, 8 μg/mL; nitrofurantoine (FT) 16, 32, 64 μg/mL; norfloxacine (NOR) 1, 8, 32 μg/mL; piperacilline/tazobactam (TZP) 4/4, 16/4, 32/4, 64/4 μg/mL; tetracycline (TE) 2, 4, 8 μg/mL; tobramycin (TM) 8, 16, 64 μg/mL; and trimethoprim/sulfamethoxazole (SXT) 1/19, 4/76, 16304 μg/mL. *E. coli* strains ATCC 25922 and ATCC 35218 from the American Type Culture Collection (ATCC) were used as controls.

### Purification of O104 LPS and K9 Capsular Antigen

Extraction and purification of O104 LPS was carried-out by the phenol-water method described by Westphal and Jann (1965) using *E. coli* strain H519 (O104:H12). K9 capsular antigen was obtained from *E. coli* strain Bi316/42 (O9:K9:H12) using a modified method of that described by Jann (1985).

### ELISA

IgG antibodies from each bovine serum were determined against O104 LPS and K9 capsular antigen by an ELISA test, as described previously (Navarro et al., 2003). The assays were placed into separate 96-well plates (MaxiSorp F96) to which, either 10 μg/mL of the O104 LPS or the K9 capsular antigen were added to each well dissolved in carbonate buffer pH 9.6. The plates were first incubated for 2 h at 37°C and then for 18–24 h at 4°C. The plates were then blocked with 200 μL of a 1.0% PBS/BSA solution and incubated for 2 h at 37°C. The plates were washed 3 times with 0.05% PBS/Tween and each well was inoculated with 100 μL of 1:1000 dilution of bovine serum sample to be tested. The plates were incubated for 2 h at 37°C and then washed 3 times with PBS/Tween 20. To each well, 100 μL of rabbit anti-IgG conjugated with alkaline phosphatase (Invitrogen) diluted 1:1000 in PBS pH 7.4 was added. Plates were then incubated for 2 h at 37°C before adding 200 mL p-nitrophenyl phosphate (1 mg/mL, Sigma) in diethanolamine buffer (pH 9.8; Sigma) to visualize the reaction, which was stopped with 25 μL of 3M NaOH. Optical density (OD) was determined in an ELISA reader (MR580 Dynatech) at 405 nm.

### Statistical Analysis

The assays were carried out in duplicate and the arithmetic means of two assays were compared by a one-tailed analysis of variance with a significance level of <0.05. A cut-off point of OD 0.7 and 1:1000 serum dilution was considered to be a positive ELISA result, as described previously (Navarro et al., 2003).

## Results

### Identification

Of the 252 fecal samples studied, 150 corresponded to Jalisco herds, and of these 58 samples corresponded to 2004, and 92 to 2005 and 2007 (46 in each year); in addition, 56 samples were from Sinaloa herds and 46 from Sonora. Of each fecal sample, five colonies were selected obtaining 1,260 cultures. In the identification of the cultures, 841 (66.7%) were identified as *E. coli* and 419 (33.3%) as other *Enterobacteriaceae* (*E. hermannii*, *Enterobacter spp.*, *Klebsiella spp.*, *Citrobacter freundii*, and *Proteus spp*.).

### Diarrheagenic Serogroups

Serotyping of the 841 cultures identified as *E. coli* strains indicated that 656 (78%) belonged to different, recognized *E. coli* serogroups and 185 (22%) were non-typable (O?). Of the strains with a recognized O group, 202 (30.8%) belonged to 21 non-pathogenic serogroups, 61 (9.3%) to 6 ExPEC serogroups, and 393 (59.2%) to 80 DEC serogroups (**Table 2** and **Supplementary Tables S1**, **S2**). Of the DEC strains, 105 (26.7%) belonged to ETEC group, 68 (17.3%) to EPEC group, 45 (11.5%) to EIEC group, 15 (3.8%) to EAEC group and 160 (40.7%) to non-O157 STEC group.

**Table 2:** Pathotypes analysis of *E*. *coli* strains isolated from dairy cows from 5 herds of three states of Mexico.

Pathogenic group	Pathotypes	Strains N (%)	Serogroups number
Non-DEC	ExPEC	61 (9.3)	6
	Others	202 (30.8)	21
DEC	ETEC	105 (26.7)	19
	EPEC	68 (17.3)	7
	EIEC	45 (11.5)	7
	EAEC	15 (3.8)	5
	STEC	160 (40.7)	42
Total	Total	656	107

*The analysis by serogroups showed that 393 of 656 (59.9%) strains belonged to 80 diarrheagenic *E*. *coli* (DEC) serogroups and 263 (40.1%) were non-DEC*.

### Serotyping and Virulence Genes

**Table 3** shows the results of the serotyping and pathogenic characteristics of the STEC strains detected in all the herds studied. The presence of *stx1* and *stx2* genes was determined in 160 STEC strains and of these, 70 (43.8%) could be grouped into 23 serogroups amplified for one or both genes, while the remaining 90 (56.3%) were negative for the presence of both genes (**Table 3** and **Supplementary Table S3**). When considering the serotypes, as well as the place and year of isolation, three Jal2004 stains were O157:H7, three O179:H8, two O146:H21 serotypes and seven belonged to different serotypes. The presence of the *stx2* gene was detected in 8 (11.4%) of these STEC strains, *stx1* + *stx2* in 7 (10%), *eae* in 3 (4.3%) and *ehxA* in 8 (11.4%) strains. For the Jal2005 samples, three STEC strains belonged to serotype O116:H21, two were O88:H25 and three strains were of different serotypes. Gene detection for these strains showed *stx1* in 1 (1.4%) strain, *sxt2* in 5 (7.1%) and *ehxA* in 3 (4.3%) strains. Finally, in the Jal2007 samples, four were O116:H16, three O121:H11, three O48:NM (non-motile), two O154:H1, two O154:H4, two O180:NM and two strains were of different serotypes. The genes detected in these STEC strains were *stx2* in 18 (25.7%) strains and *ehxA* in 9 (12.9%) strains.

**Table 3:** Association between phylogenetic groups and virulence genes of STEC strains isolated from dairy cows.

Location and year of sampling	Serotypes	Strains	Phylogenetic group^3^	Virulence genes				
				*stx1* only	*stx2* only	*stx1 + stx2*	*eae*	*ehxA*
Jal2004	O8:H8	1	B1 (15.7)	-	1	-	-	-
	O48:H8	1		-	-	1	-	-
	O58:H40	1		-	1	-	-	1
	O77:H18	1		-	1	-	-	-
	O80:H39	1		-	1	-	-	-
	O112ac:H7	1		-	1	-	-	1
	O146:H21	2		-	2	-	-	-
	O179:H8	3		-	-	3	-	2
	O157:H7^1^	3	D (4.3)	-	-	3	3	3
	O?:H48	1	E (1.4)	-	1	-	-	1
Jal2005	O3:NM	1	A (1.4)	-	-	1	-	-
	O8:H8	1	B1 (10)	-	1	-	-	1
	O88:H25	2		-	2	-	-	2
	O116:H21	3		1	1	1	-	-
	O178:NM	1		-	1	-	-	-
Jal2007	O116:H16	4	A (15.7)	-	4	-	-	4
	O154:H1	2		-	2	-	-	1
	O154:H4	2		-	2	-	-	-
	O154:H32	1		-	1	-	-	1
	O180:NM	2		-	2	-	-	-
	O121:H11	3	B1 (4.3)	-	3	-	-	3
	O48:H54	1	B2 (1.4)	-	1	-	-	-
	O48:NM	3	D (4.3)	-	3	-	-	-
Sin2005	O18ac:H7	2	B1 (27.1)	-	2	-	-	-
	O39:H21	1		-	1	-	-	1
	O88:H25	7		-	2	5	-	3
	O103:H25	1		1	-	-	-	1
	O146:H21	4		1	2	1	-	-
	O174:H28	1		1	-	-	-	-
	O175:H21	3		-	-	3	-	-
	O?:H2	3	D (5.7)	1	2			
	O?:H20	1		-	1	-	-	-
Son2005	O104:12^2^	2	A (4.3)	-	2	-	-	2
	O176:H54	1		-	1	-	-	-
	O176:H11	1	B1 (4.3)	1	-	-	-	-
	O88:H25	1		-	1	-	-	1
	O146:H21	1		-	1	-	-	-
Total, N (%)		70		6 (8.6)	46 (65.7)	18 (25.7)	3 (4.3)	28 (40)

^1^*All the O157:H7 strains were *eae*, *stx1*, *stx2*, and *ehxA* gene positives. ^2^Positive strains for the *aggR* and *aapA* genes. The remaining of the strains were negative for *aggR*, *aapA*, and *aatA* genes. ^3^Group A, *arpA*+, *chuA-*, *yjaA*+, TspE4.C2-; Group B1, *arpA*+, *chuA-*, *yjaA-*, TspE4.C2*+*; B2, *arpA-*, *chuA*+, *yjaA*+, TspE4.C2+; group D, *arpA*+, *chuA*+, *yjaA-*, TspE4.C2*+*; group E, *arpA*+, *chuA*+, *yjaA*+, TspE4.C2-. Jal, Jalisco; Sin, Sinaloa; Son, Sonora; NM, Non-motile*.

The serotypes of the Sinaloa strains isolated during 2005 included seven O88:H25 strains, four O146:H21, three O175:H2, two O non-typeable:H2, two O18ac:H7 and four strains of different serotypes. The genes detected were *stx1* in 4 (5.7) strains, *stx2* in 10 (14.3%), *stx1* + *stx2* in 9 (12.9%), and *ehxA* in 5 (7.1%) strains. In the Son2005 strains, two were O104:H12 strains and four were of different serotypes. The genes detected were *stx1* in 1 (14.4%) strain, *sxt2* in 5 (7.1%) and *ehxA* in 3 (4.3%) strains. Of the 90 STEC strains that did not amplify *stx1* or *stx2*, 13 were of serogroup O9 with different flagellar antigens (NM, H12, H21, H25, and H54) isolated from the Jal2005, Jal2007, and Sin2005 herds. These strains had genes *ehxA*, *aggR*, and *eae* when amplified (**Table 4**).

**Table 4:** Phylogenetic group and virulence genes of *E*. *coli* serogroup O9^∗^ isolated from dairy cows.

Location and year	Serotype	Strains	Phylogenetic group	*eae*	*ehx*A	*agg*R
Jal2005	O9:H12	1	B1 (7.7)	1	1	1
Sin2005	O9:H21	1	A (7.7)	1	1	1
	O9:NM	1	A (15.4)	1	1	1
	O9:H25	1		1	1	1
	O9:NM	1	B1 (23.1)	1	1	1
Jal2007	O9:H21	1		1	1	1
	O9:H54	1		1	1	1
	O9:NM	4	C (46.2)	4	4	4
	O9:H9	1		-	1	1
	O9:H12	1		-	1	1
Total, N (%)		13		11 (85)	13 (100)	13 (100)

^∗^*All strains of *E*. *coli* O9 were negative for *stx1*, *stx2*, and *aap* genes. NM, non-motile*.

### Phylogenetic Groups

Most of the non-O157 STEC strains were included in the commensal B1 (61.4%) and A (21.4%) groups, with only 17% belonging to virulent groups B2, D, and E (**Table 3**). The analysis of the *stx1* and *stx2* negative O9 strains showed that they belonged only to commensal group A (23.1%), group B1 (30.8%) and group C (46.1%) (**Table 4**).

### Bovine Serum Response to O104 LPS and K9 Capsular Antigen

A cut-off point of 0.7 OD was considered as a positive value for the ELISA test to determine a response at a 1:1000 serum dilution. This value corresponded to the mean value, plus two standard deviations when the assays were read at 405 nm, as previously reported (Navarro et al., 2003). The analysis of these assays indicated that 89 serum samples (35%) reacted with the O104 LPS and 82 (33%) with the K9 capsular antigen. The results did not show significant differences in an X^2^ analysis (**Table 5**). The serum response to these same antigens was the same for samples obtained from cows raised in the different herds (*p* > 0.05). Similar observations were obtained when a cut-off point of 0.4 DO was applied to the ELISA test. In this case, the results showed that 183 (72.6%) serum samples reacted with the O104 LPS and 200 (79.4%) with the K9 capsular antigen (*p* = 0.08).

**Table 5:** Analysis of the reactivity of serum samples from five herds of dairy cows from three states of Mexico against O104 LPS and the K9 capsular antigen.

Location and year of sampling	Samples	Antigens		
		O104 LPS	K9 capsular antigen	*p*-value^∗^
Jal2004	58	27 (47)	23 (40)	0.45
Jal2005	46	16 (35)	13 (28)	0.5
Jal2007	46	13 (28)	12 (26)	0.81
Sin2005	56	23 (41)	24 (43)	0.85
Son2005	46	10 (22)	10 (22)	1.0
Total	252	89 (35)	82 (33)	0.63

*The results are shown as the number of positive samples with the percentage in parentheses. The ELISA used a cut-off point of 0.7 at A_405_ and a serum dilution of 1:1000. ^∗^Statistical difference between the two antigens using a *X*^2^ test*.

### Antimicrobials Sensitivity

Antimicrobial susceptibility testing indicated that 52 out of 70 (74.3%) STEC strains were sensitive to all antimicrobials tested, while 18 (25.7%) were resistant to AMP, CF, CXM, TE and SXT in different combinations (**Table 6**). In difference, 92% of the non-STEC strains belonging to serotype O9, were resistant to most of the antimicrobials tested (**Table 7**).

**Table 6:** Resistance patterns of STEC strains isolated from dairy cows.

			Resistance to antimicrobials				
Herd and year	Serotype	Strains	Penicillins	Cephalosporins		Tetracycline	Trimethoprim/Sulfamethoxazole
			AM	CEP	CXM	TE	SXT
Jal2004	O8:H8	1				R	
	O58:H40	1				R	
	O146:H21	1				R	
	O146:H21	1	R	R	R	R	
Jal2005	O88:H25	2				R	
	O178:NM	1		R			
Jal2007	O116:H16	3	R				
	O116:H16	1	R			R	
	O121:H11	2	R	R		R	R
Sin2005	O174:H28	1	R	R			
	O88:H25	1	R		R		
Son2005	O146:H21	1			R		
	O104:H12	1				R	
	O104:H12	1			R		R
Total N (%)		18	9 (50)	5 (27.8)	4 (22.2)	10 (55.6)	3 (16.7)

*AM, ampicillin; CEP, cephalothin; CXM, cefuroxime; TE, tetracycline; SXT, trimethoprim/sulfamethoxazole; NM, non-motile*.

**Table 7:** Patterns of antimicrobial resistance of *E*. *coli* O9 strains.

Herd and year	Serotypes	Strains	Resistance in common to β-lactams and cephalosporins	Other antimicrobials	Total antimicrobials
Jal2005	O9:H12	1		CIP, LEV TET y SXT	4
Jal2007	O9:H21	1		AMC, TZP, CEP, and CPD	4
	O9:NM	1	AM, AMC, CEP, CZ, CXM, CPD, CAZ, CRO, and FEP		9
	O9:NM	1		TZP and TET	11
	O9:NM	1		GM and CIP	11
	O9:NM	2		GM, FOX, TET and SXT	13
	O9:H12, O9:NM, O9:H54, and O9:H25	4		TZP, GM, TM, FOX, and SXT	14
	O9:H9	1		TZP, GM, TM, FOX, CIP, TET, and SXT	16
	Total (*N* = 13)	12 (92%)			

*AM, ampicillin; AMC: amoxicillin/clavulanic acid; CEP, cephalothin; CZ, cefazolin; CXM, cefuroxime; CPD, cefpodoxime; CAZ, ceftazidime; CRO; ceftriaxona; FEP, cefepime; CIP, ciprofloxacine; LEV, levofloxacin; TE, tetracycline; SXT, trimethoprim/sulfamethoxazole; TZP, piperacilline/tazobactam; GM, gentamicin; FOX, cefoxitin; TM, tobramycin; NM, non-motile*.

## Discussion

Although diarrheal disease mortality has decreased significantly over the past 15 years, morbidity due to this problem continues to be high (Troeger et al., 2017). Although currently available vaccines against some of the most frequent viral pathogens have shown their capacity to reduce mortality, the search for the source of these infections is essential for their final control. Water and food sampling constitute the most accessible way to look for these sources. That is the reason why we conducted a sequential sampling of feces and sera of cows belonging to herds in three of the largest cattle-rearing regions in Mexico. Over a number of years, the search for putative pathogenic bacteria in these samples has shown the presence of putative pathogenic strains able to cause disease in humans. Around 40% of the putative pathogenic *E. coli* found in the sample animals belonged to non-O157 STEC strains. This has also been found in previous studies conducted in other parts of the world (Hussein and Sakuma, 2005; Monaghan et al., 2011). The isolation of O157:H7 strains is interesting since, to date, there have been no reported outbreaks of HC and HUS in Mexico associated with these type of isolates. Their presence in these herds is a wake-up call to continue this surveillance since it is clear that cows from these herds harbor both O157:H7 and non-O157 strains that could be the source of a human outbreak.

ETEC strains were the second most frequent DEC group of strains found in the present study. ETEC strains have been associated worldwide with cases of diarrhea in pigs, calves, and rabbits and are an important cause of diarrhea in children in low and middle income countries (Shaheen et al., 2004; Wennerås and Erling, 2004; Qadri et al., 2005; Troeger et al., 2017) and in travelers to these areas of the world (Shah et al., 2009; Croxen et al., 2013). In the present study, we were able to identify ETEC strains belonging to 19 different serotypes, all of them previously reported from other parts of the world (Wolf, 1997; Shaheen et al., 2004).

The lack of clinical symptoms in the cows harboring strains belonging to pathogenic STEC types O157:H7 and O104:H12 confirm previous findings and indicate the risk for humans coming in contact with these animals or their feces (Quilliam et al., 2012; Shridhar et al., 2018). However, there is no clear explanation as to why these fully pathogenic strains found in cows have not been able to cause human disease. Strains belonging to serotype O104:H7 have been previously found in healthy Mexican lambs (Enriquez-Gómez et al., 2017). No human cases associated with these strains have been reported to date, either. In the present study, strains of serotype O104:H12 were also isolated during 2005 from the herd raised in Sonora. These strains carried *stx2, aggR*, and *aatA* genes, similar to the O104:H4 strains that caused the HC and HUS outbreak in Germany (Scheutz et al., 2011). In addition to the O104 isolates, cows from herds in the three States also carried strains belong to the O88:H25 serotype that had been previously reported as belonging to STEC and EPEC groups (Irino et al., 2005; Blanco Crivelli et al., 2018). Gene analysis of these O88:H25 strains indicated that they carried the *stx2* gene, making them potentially virulent non-O157 STEC strains.

The higher percentage of strains belonging to phylogenetic B1 groups found in the present study is similar to previous findings from other parts of the world where these B1 strains have been isolated from herbivorous animals, such as goats, lambs and cows (Baldy-Chudzik et al., 2008; Ziebell et al., 2008; Carlos et al., 2010).

Observations in our laboratory over the past 20 years have shown that *E. coli* of serotype O104 and STEC O9:K9 is not an infrequent finding in human fecal samples (unpublished results). This has led to our continued interest in studying the cross reactions that these two groups of strains have with each other and, as has been previously reported, to both the O104 LPS and the K9 capsular antigen (Kogan et al., 1992; Balabanova et al., 2013). Serum from the cows raised in the five herds tested showed a similar level of response, which signaled a common type of cross-reaction in the serum samples of all the animals tested. The development of antibodies (IgG) to both the O104 LPS and K9 capsular antigen in the same animal indicates the possibility that these animals have a gradual and low intensity repeated exposure to *E. coli* O104 strains. Another factor that may give rise to the presence of anti-O104 LPS and anti-K9 capsular antigen antibodies in the serum of these animals could be colonization by specific *E. coli* O9:K9 strains. These findings could be helpful to develop better oriented vaccines that might protect humans by inhibiting their capacity to colonize animal reservoirs. The implication for animal vaccines against O157:H7 is obvious (Navarro et al., 2016).

In terms of our findings concerning the frequency of antimicrobial resistance, the STEC Mexican strains, including the isolated O157:H7, showed a much lower presence of this characteristic than the one described in other countries (Schroeder et al., 2002). On the other hand, the results of these assays in the O104:H12 strains indicated a similar frequency of antimicrobial resistance to one (CEP) or two (TE and SXT) antimicrobials, as previously reported by Schroeder et al. (2002), but with different patterns to the O104:H4 strains isolated during the epidemic outbreak in Germany (Frank et al., 2011). Interestingly, multiple antimicrobial resistance was found in O9 strains we studied, which is in line with reports from previous studies for *E. coli* strains isolated from extraintestinal sources (Johnson et al., 2004). All these results continue to point to the use of antimicrobials as part of the food given to farm animals, which needs to be addressed as one of the main causes for the increase in multiple antimicrobial resistance in human infections (McEwen and Fedorka-Cray, 2002; Tadesse et al., 2012).

The presence of *E. coli* strains belonging to two of the most important human DEC pathotypes isolated from the feces of the animals studied indicates their importance as a potential source of virulence strains able to cause human infections. The participation of food, water and other environmental sources, as well as direct human contact with animals has been well documented in terms of infections by *E. coli* strains belonging to the O157:H7 serotype (Caprioli et al., 2005; Quilliam et al., 2012). In addition, the spread of pathogenic bacteria could be enhanced by the use of fresh manure to fertilize soil for the purpose of growing vegetable crops (Durso et al., 2011). The results of the present study clearly support these findings and point to the need for continued surveillance of these putative sources of human disease.

Finally, the presence of multiresistant strains as part of the intestinal microbiota of cattle could be the source of difficult-to-treat extraintestinal infections caused by *E. coli* strains that have received these genes through horizontal transfer in the intestinal environment (Penders et al., 2013). Bearing this in mind, our study reveals the importance of epidemiologic surveillance of the natural reservoirs of DEC strains in groups of animals used for human consumption.

## Author Contributions

AN and CE designed the research. PC-S, AT, AG, SD, and MDC performed the sampling design and experiments. AN, AC, and CE analyzed the data and wrote the paper.

## Conflict of Interest Statement

The authors declare that the research was conducted in the absence of any commercial or financial relationships that could be construed as a potential conflict of interest.
